# A 3D‐Engineered Conformal Implant Releases DNA Nanocomplexs for Eradicating the Postsurgery Residual Glioblastoma

**DOI:** 10.1002/advs.201600491

**Published:** 2017-03-30

**Authors:** Yuan Yang, Ting Du, Jiumeng Zhang, Tianyi Kang, Li Luo, Jie Tao, Zhiyuan Gou, Shaochen Chen, Yanan Du, Jiankang He, Shu Jiang, Qing Mao, Maling Gou

**Affiliations:** ^1^ State Key Laboratory of Biotherapy and Cancer Center West China Hospital Sichuan University and Collaborative Innovation Center of Biotherapy Chengdu P. R. China; ^2^ Department of Neurosurgery West China Hospital Sichuan University Chengdu P. R. China; ^3^ School of Materials Science and Engineering Sichuan University Chengdu Sichuan 610065 P. R. China; ^4^ Department of Nanoengineering Institute of Engineering in Medicine 245B SME Building MC 0448 University of California San Diego 9500 Gilman Drive La Jolla CA 92093 USA; ^5^ Department of Biomedical Engineering Tsinghua University School of Medicine Beijing P. R. China; ^6^ State key laboratory for manufacturing systems engineering Xi'an Jiaotong University Xi'an 710049 China

**Keywords:** 3D printing, conformal implants, cancer gene therapy, nanoparticles

## Abstract

Gene therapy has great promise for glioblastoma treatment; however, it remains a great challenge to efficiently deliver genes to the brain. The incomplete resection of glioblastoma always leads to poor prognosis. Here, a 3D‐engineered conformal implant for eradicating the postsurgery residual glioblastoma is designed. This implant is constructed by 3D‐printing technology to match the tumor cavity and release an oncolytic virus‐inspired DNA nanocomplex to kill glioblastoma cells through apoptosis induction. Meanwhile, a 3D‐engineered subcutaneous glioblastoma xenograft is built to mimic the resection tumor cavity in mice. Insertion of the implant into the glioblastoma resection cavity efficiently delays tumor recurrence and significantly prolongs overall survival. This study provides a proof‐of‐concept of glioblastoma therapy using a conformal implant that releases oncolytic DNA nanocomplexs. This strategy can lead to the development of future precision therapy for eradicating postsurgery residual tumors.

## 1 Introduction

Surgical resection is the primary choice for clinically treating glioblastoma multiforme (GBM).^1^ However, postoperative glioma recurrence always occurs from residual cancer tissues, causing patient death.^2^ Currently, patients who suffer from GBM have a median survival period of only 14.6 months, and some have a 5 year survival rate of less than 10%.^3^ Therefore, novel drugs that can efficiently eliminate residual cancer cells are desired for prolonging the overall survival of GBM patients. Gene therapy is a promising approach for treating cancer.^4^ Vesicular stomatitis virus (VSV) can dissolve tumors, and its matrix protein (VSVMP) can independently cause considerable tumor cell cytopathogenesis in the absence of other VSV components.^5^ Recently, we found that plasmid DNA encoding VSVMP (pVSVMP) can be used to eliminate cancer cells and induce an anticancer immunity response,^6^ thereby demonstrating potential for application in cancer gene therapy. However, delivering therapeutic genes to gliomas remains challenging because of the blood–brain barrier,^7^ meanwhile, these genes must be able to withstand the substantial dynamic forces in the brain caused by cerebral spinal fluid flow.^8^ Thus, conventional gene delivery strategies via the intravenous administration or local injection of gene therapy solutions are limited for treating conditions involving the central nervous system.^9^ Despite these challenges, glioma patients who undergo surgical tumor resection are left with a residual tumor cavity, which provides a place for the local delivery of genes to eliminate residual glioma cells.

**Figure 1:**
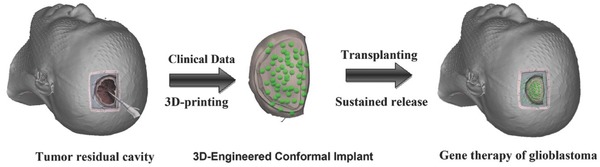
Schematic diagram of an implant surgically situated in a tumor residual cavity. The 3D data of the tumor residual cavity (left) could be acquired through intraoperative CT/MRI scans, and then the 3D implant (middle) could be fabricated and implanted into the patient's tumor cavity (right).

Herein, we engineered a 3D therapeutic device (**Figure** **1**) that can match postoperative tumor cavities and release DNA nanocomplexs to eliminate residual glioma cells. The DNA nanocomplexs are composed of pVSVMP and degradable heparin‐polyetherimide (HPEI) nanogel particles and can efficiently eliminate glioma cells after transfection. In this study, we fabricated the implant and tested its cytotoxicity against U87 human glioblastoma cells both in vitro and in vivo. Furthermore, we developed a novel method for introducing the nanocomplexs into a tumor cavity after glioblastoma debunking surgery. This engineered implant could be an effective therapeutic method for treating glioblastoma‐adjacent areas.

## 2 Results and Discussion

**Figure 2:**
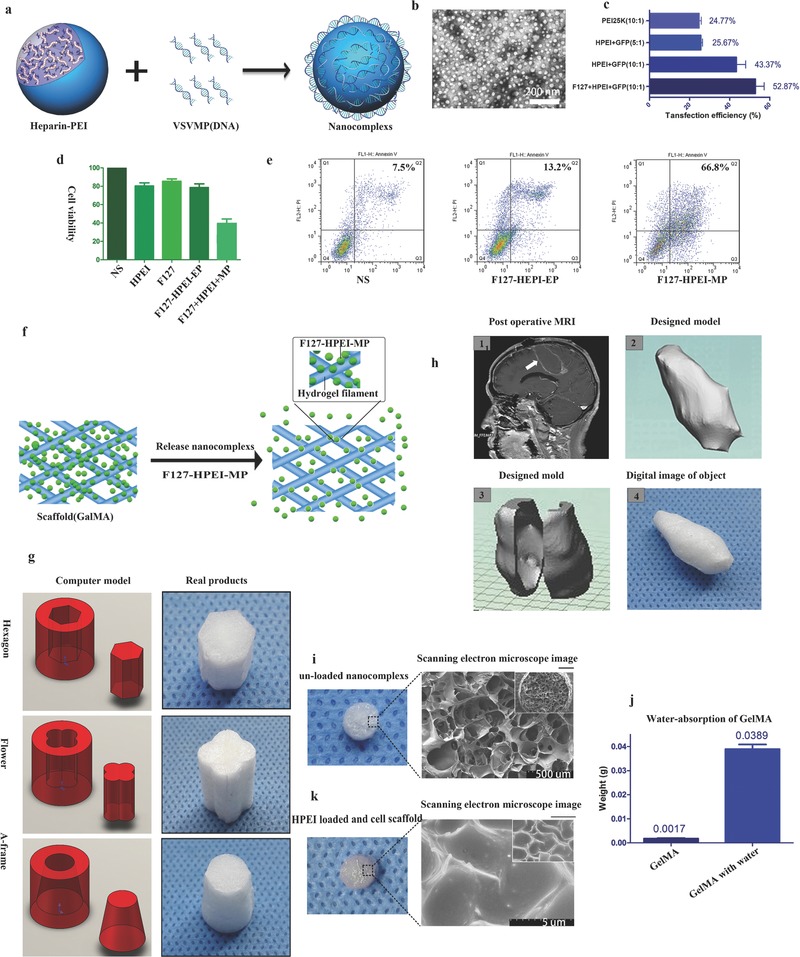
a) Preparation scheme of HPEI nanogels. b) TEM images of HPEI nanogels. Scale bar, 200 nm. c) Transfection efficiency of the PEI25K, HPEI nanogels+GFP (5:1/10:1), and F127+HPEI nanogels+GFP (10:1). The amount of pGFP was maintained at 2 µg per well. Flow cytometry (Epics Elite ESP, USA) was used to determine the percentage of transfected cells. d) MTT assays were used to compare cell viability of the NS, HPEI, F127, HPEI‐F127‐EP (empty plasmid), and HEPI‐F127‐MP groups; the ratio of HPEI and EP/MP was 10:1. e) Cell viability as determined by Annexin V‐FITC assays comparing the NS, F127‐HPEI‐EP, and F127‐HPEI‐VSVMP groups. f–h) Schematic illustrating the synthesis of the 3D implant. f) A certain amount of a gene solution was dropped onto the scaffold to form a gene composite, and the DNA was subsequently released in a sustained manner from the scaffold. g) Representation of a 3D conformal implant designed using data from a postoperative glioma surgery patient. h) Illustration and images of different composite shapes synthesized using GelMA. i) SEM image of a scaffold (GelMA) without particles. k) SEM image of a scaffold (GelMA) loaded with HEPI nanoparticles. j) The water‐absorption ability of GelMA.

As illustrated in **Figure** **2**a, HPEI nanoparticles were prepared to transfer pVSVMP into U87 glioma cells. These nanoparticles were monodispersed (polydispersity index = 0.156) with a hydrogel structure and a mean particle size of ≈75 nm in water and 25 nm when dry (Figure 2b). The HPEI nanoparticles had a cationic surface (zeta potential = 27.1 mV) and could bind DNA (Figure S1a,b, Supporting Information). The capacity to bind DNA of HPEI was assessed by an agarose gel electrophoresis retardation assay. HPEI could completely retard the electrophoretic mobility of DNA at appropriate ratio of nitrogen to phosphate (N/P level 8, Figure S1c, Supporting Information). To improve the transfection efficiency of HPEI, Pluronic F127 was added to the DNA complexes. As shown in Figure 2c, compared with HPEI and PEI25K, the F127‐HPEI composites exhibited a ratio increased by ≈20% (Figure S1d, Supporting Information). The ability of F127 to improve the transfection efficiency may have been because Pluronic is a nonionic surfactant that can facilitate passage through the cell membrane, thereby improving the cellular uptake of DNA Nanocomplexs.^10^

To determine the anticancer effect of F127‐HPEI‐VSVMP in vitro, an 3‐(4,5)‐dimethylthizol‐2‐yl)‐2,5‐diphenyltetrazolium bromide (MTT) assay was used to evaluate cell viability. As shown in Figure 2d, the MTT results were analyzed after U87 cells were incubated with F127‐HPEI only or F127‐HPEI‐VSVMP complexes for 48 h. U87 cells were efficiently inhibited by the F127‐HPEI‐VSVMP complexes, while the cells treated with F127‐HPEI or empty plasmid (F127‐HPEI‐EP) complexes maintained high viability. These results showed that the F127‐HPEI‐VSVMP complexes exerted an anticancer effect on U87 cancer cells with a low vehicle cytotoxicity. Moreover, as presented in Figure 2e, the flow cytometry results showed a greater proportion of apoptotic cells (66.8%) in the F127‐HPEI‐VSVMP group than in the other groups (normal saline 7.5%, null‐F127‐HPEI 13.2%).

To locally delivery the nanocomplexs for treating postoperative glioblastoma patients, we prepared nanocomplexs‐loaded conformal scaffolds for implantation. The preparation scheme of fabricating the implants is presented in Figure 2f. First, we engineered a gelatin methacrylamide (GelMA) scaffold designed depending on the required shape. Then, we dropped solution containing the F127‐HPEI‐VSVMP complexes onto the printed scaffold, resulting in an oncolytic Implant.

Using this customizable 3D fabrication method, we can design implants in any shape (Figure 2g). To assess the feasibility of this method, we used magnetic resonance imaging (MRI) data to fabricate a conformal 3D implant according to the shape of the postoperative cavity of a glioma patient (Figure 2h). For the in vitro and in vivo testing of the anti‐tumor ability of the implant, we chose a cylindrical shape for the Implant DNA carrier (Figure 2i). A 7% GelMA solution was used to form the designed shape with a diameter of 4 mm and a height of 1.5 mm. Digital and scanning electron microscopy (SEM) images of the composite are shown in Figure 2i. This GelMA cylinder had a net structure, which may be responsible for preventing the nanocomplexs from being washed out by the cerebral spinal fluid. The implant could absorb water up to 20 times its own weight (Figure 2j), exhibiting the high encapsulation efficiency of the F127‐HPEI‐EP nanogels. SEM was used to estimate the distribution of Nanocomplexs within the Implant (Figure 2k). The HPEI/DNA particles encapsulated within the GelMA appeared to be well dispersed, and the size distribution of the particles was similar to the average diameter determined by transmission electron microscopy.

**Figure 3:**
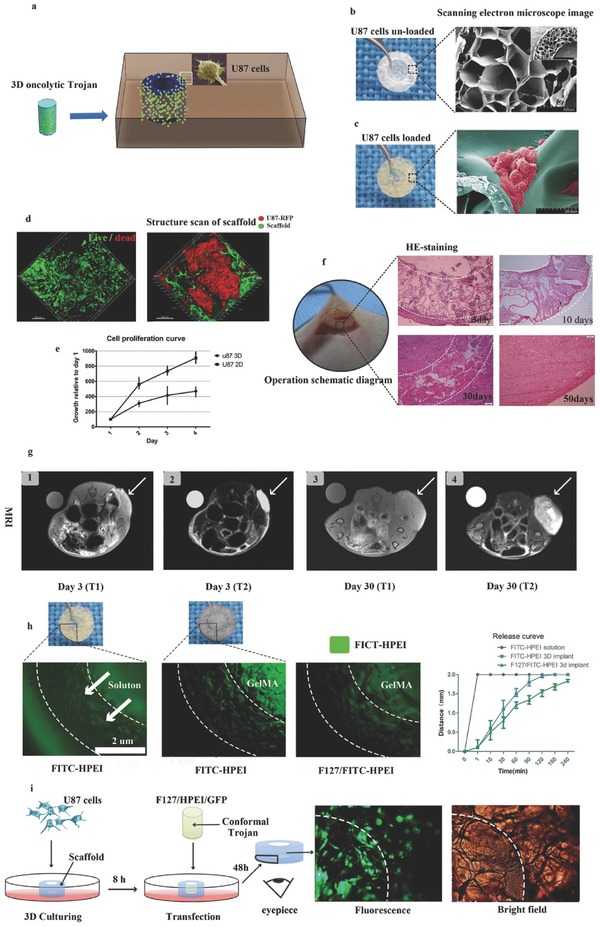
a) Schematic diagram of an implant surgically situated in a tumor cavity. The tumor cavity scaffold used in our study was printed with GelMA and U87 cells loaded in vitro for 48 h; then, the matched 3D conformal implant with F127‐HEPI‐MP was transferred into the tumor cavity of a nude mouse to mimic the designed treatment strategy. b) Digital (left) and SEM images (right) of the printed tumor cavity. Scale bar, 200 µm. c) Digital (left) and SEM images (right) of the printed tumor cavity with U87 cells cultured 48 h. Scale bar, 20 µm. The cells proliferated on the scaffold in a 3D manner. d) Live/Dead assay (left) showing that most of the U87 cells were alive after being embedded in the scaffolds for 48 h in vitro. U87 cells labeled with mCherry were embedded in scaffolds fluorescently tagged with FITC (right). Confocal microscopy showing that the U87 cells proliferated in clustered in the scaffold like in vivo. e) Cell proliferation curves of U87 cells cultured in 3D and 2D. f) Implementation of the approach: the mimic tumor cavity with U87 cells embedded for 48 h was implanted into the nude mice (left); H&E staining showing gradual glioma recurrence and scaffold biodegradation in vivo. g) MRI scans of the implanted scaffold with U87 cells: (1) T1 sequence of the tumor on day three after implantation surgery; (2) T2 sequence on day three; (3) T1 sequence on day thirty; (4) T2 sequence on day thirty. The arrow indicates the implant. h) Digital and corresponding fluorescence images of the in vitro assay used to quantify F127‐HPEI nanogel release; the results showed slow and steady release. Quantification of the distance of viable HPEI‐FITC‐labeled cells released from the implants at the indicated time points. i) Schematic and corresponding fluorescence images of the in vitro assay showing that the F127‐HEPI‐GFP composites could efficiently transfer into U87 cells.

To maximally simulate a GBM resection, as well as validate the cytotoxicity of the implant to human U87 cells in vitro, we engineered a 3D GelMA scaffold in the shape of a resection tumor cavity.^11^ Then, we cultured human U87 cells on the scaffold to replicate the glioma residual cavity ex vivo, and the shape‐matched implant was then inserted into the glioma residual cavity. An image of ischemic glioma in vitro after using the implant for gene therapy is shown in **Figure** **3**a. A 5% GelMA solution was used to achieve the desired shape of the tumor cavity with an inner diameter of 4 mm, an outer diameter of 8 mm and a thickness of 0.5 mm (Figure 3b). Digital and SEM images of the tumor cavity scaffold are shown in Figure 3b. The scaffold had a relatively large porous network that could support cell proliferation and migration. To observe cellular morphology on the scaffold, SEM was used to image U87 cells cultured on the 3D scaffold. These images showed that the U87 cells on the 3D scaffold had a more complicated cellular morphology and tumor microenvironment than did cells cultured in 2D (Figure 3c, Figure S1e,f, Supporting Information). Furthermore, after U87 human GBM cells were transduced with a lentivirus (GFP), confocal microscopy confirmed that cells grew in clusters in the GelMA (Figure 3d, right).

To determine whether U87 cells could be cultured on GelMA, a Live/Dead cell viability assay was conducted after U87 cells were cultured for 72 h on the engineered scaffold; subsequent confocal microscopy demonstrated that only two or three cells were dead (Figure 3d, left). The MTT cell proliferation assay showed that the growth of U87 cells cultured in vitro in 2D was significantly faster than that of cells cultured in 3D (Figure 3e). Then, to determine whether U87 cells cultured on a scaffold could mimic glioma recurrence, after 24 h of incubation, a scaffold containing 2 × 10^6^ U87 cells was directly implanted into the subcutaneous space of a null mouse (Figure 3f). Tumor recurrence occurred thirty days after introducing the residual tumor cell model into the mouse; most importantly, hematoxylin and eosin (H&E) staining of the tumor tissue demonstrated that most U87 cells in the supporting biomaterial were viable (Figure 3f). The U87 cells could migrate from the GelMA and infiltrate surrounding tissues, simulating tumor recurrence. We also assessed the status of scaffolds in vivo using MRI (Figure 3g). In accordance with the H&E results, Figure 3, the scaffold gradually degraded, and the tumor recurred, supporting the hypothesis that propagating cancer cells are released over time.

This manufactured implant was designed to release apoptosis genes, to determine whether the nanocomplexs could be released from the engineered implant, the HPEI was conjugated with fluorescein isothiocyanate (FITC). Subsequently, we bedded the implant into the simulated tumor cavity in vitro, as shown in Figure 3h. The Implant was observed by fluorescence microscopy to determine whether the F127‐HPEI‐VSVMP could slowly release the genetic material; in these images, fluorescence indicates the presence of HPEI. As expected, the material in the solution‐only group reached the cavity edge immediately (Figure 3h), while the material in the FITC‐HPEI with GelMA implant group infiltrated outward slowly; when 5% (w/v) F127 was added, the HPEI was released even more slowly. The implant scaffold, GelMA, played a major role in the slow release of FITC‐HPEI, and because of nonspecific interactions, such as electrostatic interactions between the positively charged nanocomplexs, and the hydroxyl and carboxyl groups of the hydrogel which may impart a net negative charge, as well as the precipitation of DNA complexes into the hydrogel substrate, resulting in relative slow‐release in the F127‐HPEI‐FITC group. To verify that the gene therapy products released from the Nanocomplexs were functional, we used F127‐HPEI‐green fluorescence protein (GFP) implants to transfer the GFP gene into U87 cells previously cultured on the engineered scaffold. As shown in Figure 3i, after implanting the 3D composites into the scaffolds and culturing for 48 h, many U87 cells expressed green fluorescence.

HPEI has been shown to be effective for gene delivery due to its ability to condense DNA.^12^ Our previous study showed that HPEI could be degraded as low molecular weight PEI and excreted via the urine.^6^ VSVMP is one of the five structural proteins (N, P, M, G, and L) of this virus, and it can arrest host cell mRNA expression, leading to systematic cellular breakdown through apoptosis.^5^, ^13^ The application of VSVMP solves the potential biohazard of using an oncolytic virus as an anticancer treatment in the central nervous system.^14^ To develop a gene therapy protocol for glioblastoma, we attempted to use HPEI‐VSVMP nanocomplexs to eradicate U87 human cells in vitro and in vivo, and the results showed that pVSVMP delivery significantly inhibited the U87 cells, indicating that HPEI‐VSVMP nanogels have potential clinical applications in treating glioblastoma.

**Figure 4:**
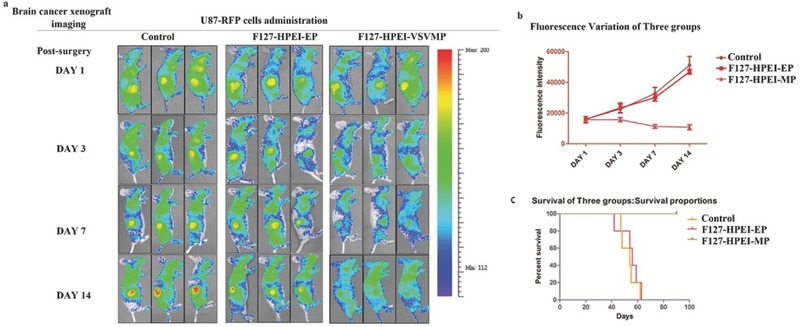
F127‐HEPI‐MP nanocomplexs robustly expand in tumor tissue, where they reduce residual disease and relapse. a) Longitudinal in vivo bioluminescence imaging of U87 cells retrovirally transduced with m‐Cherry. b) mCherry‐luc signal intensities after implant transfer; each line represents a group (control, HEPI‐EP, or F127‐HPEI‐MP). c) Kaplan–Meier curves of animal survival following 3D conformal gene therapy.

The antitumor efficiency of the implant was tested using a U87 tumor resection mouse model, which mimics the subtotal resection state of brain cancer patients. The study design included three groups: group 1, treated with an implant containing F127‐HPEI‐MP; group 2, treated with an implant containing F127‐HPEI‐EP; and group 3, treated with an implant containing an equal volume of phosphate‐buffered saline (PBS) as the negative control. U87 cells were labeled with fluorescein (U87‐mCherry) as a detection signal for bioluminescence imaging (**Figure** **4**a). First, the engineered tumor cavity scaffolds were manufactured and implanted with 2 × 10^6^ U87‐mCherry cells; then, the luciferase‐expressing U87 cells were incubated for 24 h on the scaffold before implantation. Subsequently, the engineered tumor cavity with U87 cells was transplanted into a femoral subcutaneous space simultaneously with the implant. Bioluminescence imaging was performed for observations on 14 sequential days, and the fluorescence intensity was significantly different between the treatment and nontreatment groups (groups 2 and 3) (Figure 4b). After 3 d of treatment, only nonspecific fluorescence was observed in the F127‐HPEI‐VSVMP group, while the fluorescence signal intensity continuously increased greatly over time in those groups not exposed to VSVMP. Fourteen days after implantation, the fluorescence intensity of the control group was increased by 3‐fold compared with that at day 1, and the fluorescence intensity of the F127‐HPE‐EP group increased slightly slower than did that of the control group. This result can be explained by F127‐HPEI‐EP potentially inducing a small degree of U87 cell death during the transplantation. We also measured the overall survival of the mice in the groups and constructed Kaplan–Meier curves. We found that the control and F127‐HPEI‐EP groups had no significant differences in survival (median survival: 56 versus 54 d, respectively, Figure 4c); however, the F127‐HPEI‐VSVMP treatment group showed a significant advantage in survival. As expected, the F127‐HPEI‐VSVMP complexes produced anti‐tumor benefits and prevented tumor recurrence in vivo.

The standard treatment protocol for patients with glioblastoma is maximum surgical tumor debunking and adjuvant chemo‐radiotherapy;^5^, ^15^ however, almost all preclinical models have been focused on treating established solid tumors.^5^, ^16^ In this study, we engineered a 3D glioblastoma resection model for in vitro and in vivo research. Moreover, we integrated fluorescent and bioluminescent markers and extensive optical imaging to simultaneously confirm the establishment of the resection model. Confocal microscopy and SEM showed that the resulting cellular morphology and growth patterns in the resection model were different from those of 2D cultures but similar to those of glioblastoma in vivo. This GBM resection model shows more clinical relevance than current xenograft models mimicking solid tumors; moreover, this model could be used to estimate drug susceptibility or test other anticancer drugs for glioblastoma.

Currently, the most widely used locally delivered polymer implant for brain cancer is 1,3‐bis[2‐chloroethyl]‐1‐nitrosourea (BCNU), also known as the Gliadel wafer,^17^ which is a polymer containing 7.7 mg of carmustine or BCNU that was first applied in clinical practice in 2002. The biodegradable wafer attaches to residual glioma tissues and continuously releases chemotherapy drugs in the surgical cavity. However, the effectiveness of the Gliadel wafer did not meet clinical expectations as the usefulness of the wafer was limited by its poor ability to conform to tumor walls and tumor resistance; these are all major challenges faced by local delivery approaches. We suggested that this approach of using 3D conformal implant to induce residual tumor cell apoptosis is previously unknown.

Our current work reveals an engineered implant that exerted promising therapeutic effects in vivo and in a mouse model of GBM resection. To the best of our knowledge, this is the first attempt to integrate 3D printing technology and gene therapy to treat postoperative residual glioblastoma. The design and fabrication methods for producing the implant have potential for clinical applications. This implant is fabricated to match the tumor cavity, thereby achieving a high contact area for releasing nanocomplexs. Furthermore, the scaffold of the implant is a porous structure that can prevent DNA complexes from being rapidly washed out by the cerebrospinal fluid. Moreover, oncolytic HPEI‐MP Nanocomplexs retained in the tumor cavity significantly prolonged mouse survival, demonstrating the promising antitumor efficacy and safety of this implant in treating glioblastoma.

## 3 Conclusion

In conclusion, we engineered a 3D conformal implant for eradicating the postoperative residual glioblastoma. This implant can match the tumor cavity and release VSV‐inspired pVSVMP nanocomplexs to eliminate glioblastoma cells by inducing apoptosis. Transplantation of the implant into a glioblastoma resection cavity can efficiently delay tumor recurrence and significantly prolong overall survival. This study provides a proof‐of‐concept for glioblastoma gene therapy using a 3D engineered nanocomplexs implant. This work could inspire the development of future gene therapies for glioblastoma and other postoperative residual tumors.

## 4 Experimental Section

*Fabrication of GelMA*: GelMA was prepared by reacting type A gelatin (Sigma–Aldrich, St. Louis, Missouri, USA) with methacrylic anhydride (Sigma–Aldrich) at 50 °C for 1 h. Briefly, methacrylic anhydride was added dropwise into a gelatin solution 10% (w/v) in PBS and stirred constantly for 1 h; then, the fivefold addition of warm (40 °C) PBS was used to stop the reaction. The mixture was dialyzed against distilled water for 7 d to remove methacrylic acid and anhydride, freeze‐dried, and then stored at −20 °C until use.

*Fabrication of 3D Conformal Implant and Cell Scaffolds by GelMA*: The desired models for the implants and scaffolds were previously fabricated by 3D printing. The freeze‐dried GelMA was mixed at 7% or 5% with MiniQ water at 70 °C until fully dissolved; then, 0.5% sodium dihydrogen phosphate and 0.1% ammonium sulfite was added to the dissolved GelMA. The 7% GelMA solution for the implants and the 5% GelMA solution for the scaffolds were added to the appropriate models before gelation, and these GelMA models were allowed to crosslink for 1 night at −20 °C. They were then freeze‐dried and carefully removed from the GelMA models. The implants and scaffolds were exposed to ultraviolet light for 24 h before use.

*Synthesis of HPEI Nanoparticles*: HPEI was prepared using amide bond formation between the amine groups of PEI and the carboxyl groups of heparin. Briefly, 50 mg of heparin was dissolved in an 2‐(N‐morpholino)ethanesulfonic acid monohydrate solution buffer (0.05 m, 100 mL); then, 20 mg of 1‐ethyl‐3‐(3‐dimethylaminopropyl)carbodiimide and 30 mg of N‐hydroxysiccinimide were added to this solution to activate the carboxylic acid groups of heparin for 2 h. The activated heparin solution was dropped into a PEI2K solution (7.5 mg mL^−1^, 20 mL) under constant stirring. This reaction was carried out at room temperature overnight. The resulting HPEI nanoparticles were dialyzed in distilled water for three days, filtered by a syringe filter (Millex‐LG, Millipore Co., Billerica, MA), adjusted to a concentration of 1 mg mL^−1^, and stored at 4 °C until future use.

*Fabrication of 3D Conformal Gene Therapy Composite*: The pVAX plasmid (Invitrogen, San Diego, CA) expressing wild‐type VSVMP (i.e., pVSVMP) was constructed in the laboratory as described in the previous reports. As a control, a pVAX plasmid without VSVMP cDNA was used as an empty vector (i.e., pEP). HPEI–DNA complexes were prepared by an electrostatic method in deionized water. An HPEI solution (3 µg, 3 µL, 1 mg mL^−1^) was mixed with 1 µg of pVAX/GFP plasmid, pVAX/null plasmid, or pVAX/VSVMP plasmid, followed by incubation for 30 min at room temperature. Then, an F127 solution was added to the HPEI/DNA solution on ice (F127:HPEI:DNA, 0.9:15:3 µg), and the resulting solution was dropped slowly onto the printed implants to fabricate 3D conformal gene therapy composites.

*Implantation of Cell Scaffolds and 3D Conformal Composites*: All animal procedures followed the institutional laboratory animal research guidelines and were approved by the Sichuan University Experimental Animal Center. Animals were housed in the animal facility of the State Key Laboratory of Biotherapy and Cancer Center. Female BALB/cJ mice (4–6 weeks old) were obtained from the laboratories. Thirty minutes before surgery, 2 × 10^6^ U87 cells were implanted in the scaffolds, and 3D conformal composites were prepared. The animals were anesthetized using 60 µL of 10% chloral hydrate. The initial incision was ≈1–1.5 cm; this was then widened and separated using eye scissors to allow for scaffold implantation. The U87 cell‐loaded scaffold was inserted into the subcutaneous space at least 0.5 cm away from the incision. If needed, the 3D conformal composite or 3D implant was matched to the scaffold first and then inserted into the subcutaneous space, in accordance with the experimental group.

## Supporting information

Supplementary
