# Supplementary material for: A 3D‐Engineered Conformal Implant Releases DNA Nanocomplexs for Eradicating the Postsurgery Residual Glioblastoma
Source: Adv Sci (Weinh). 2017 Mar 30;4(8):1600491. doi: 10.1002/advs.201600491 (PMC5566247; doi:10.1002/advs.201600491)

Copyright WILEY-VCH Verlag GmbH & Co. KGaA, 69469 Weinheim, Germany, 2017.

ADVANCED SCIENCE
Open Access

Supporting Information

for *Adv. Sci.*, DOI: 10.1002/advs.201600491

A 3D-Engineered Conformal Implant Releases DNA Nanocomplexs for Eradicating the Postsurgery Residual Glioblastoma

Yuan Yang, Ting Du, Jiumeng Zhang, Tianyi Kang, Li Luo, Jie Tao, Zhiyuan Gou, Shaochen Chen, Yanan Du, Jiankang He, Shu Jiang, Qing Mao, and Maling Gou**

Supporting Information

Supporting Figure 1. a. Size distribution spectrum of HPEI nanogels; b. zeta potential spectrum of HPEI nanogels; c. the DNA-binding ability of HPEI nanogels as determined by a gel retardation assay; d. fluorescence image of U87 cells incubated with F127-HPEI-GFP (10:1); e. confocal microscopy image of U87 cells after 3D culture; f. SEM image of U87 cells after 2D culture.

Sup. Figure 1.

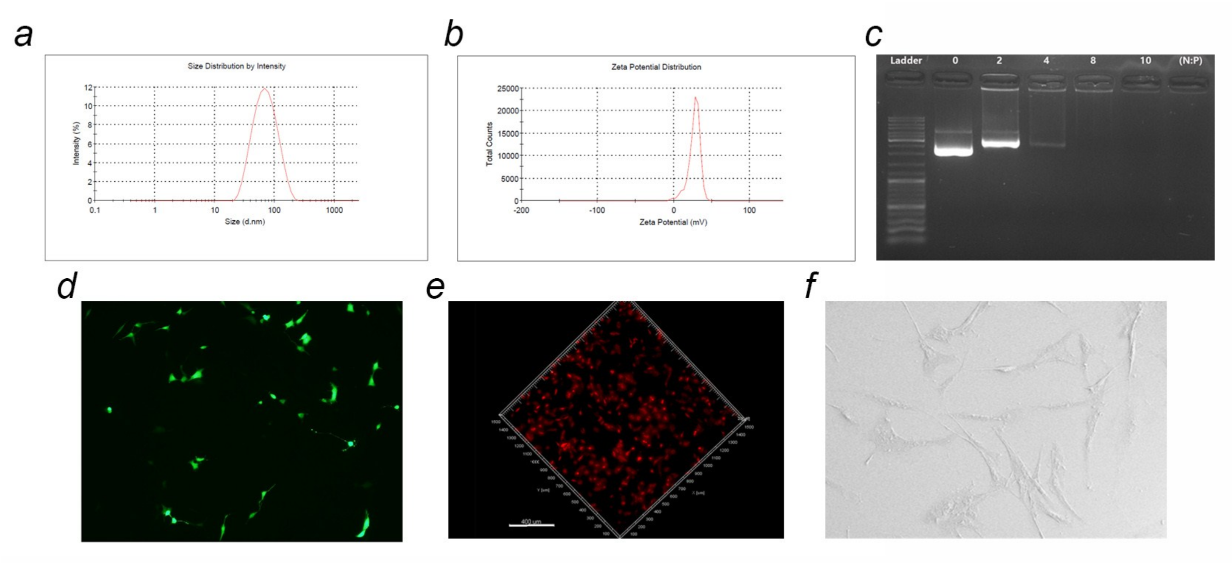

Supplement: Supplementary file 1 — Supplementary [file ADVS-4-na-s001.pdf]
